# *Cassiopea xamachana* microbiome across anatomy, development, and geography

**DOI:** 10.1371/journal.pone.0319944

**Published:** 2025-04-11

**Authors:** Allison H. Kerwin, Aki Ohdera, Juliet Bier, Devon Goodman, Marta Mammone, Victoria Sharp, Alesandra Echeandía, Mónica Medina

**Affiliations:** 1 Department of Biology, McDaniel College, Westminster, Maryland, United States of America; 2 Department of Biology, Pennsylvania State University, University Park, Pennsylvania, United States of America; Tsinghua University, CHINA

## Abstract

The upside-down jellyfish holobiont, *Cassiopea xamachana*, is a useful model system for tri-partite interactions between the cnidarian host, the photosymbiont, and the bacterial microbiome. While the interaction between the host and photosymbiont has been well studied, less is understood of the associated bacterial community. To date, the bacterial microbiome of wild *C. xamachana* has remained largely uncharacterized. Thus, wild medusae (n=6) and larvae (n=3) were collected from two sites in the Florida Keys. Bacterial community composition was characterized via amplicon sequencing of the 16S rRNA gene V4 region. The medusa bacterial community was dominated by members of the Alphaproteobacteria and Gammaproteobacteria, while Planctomycetota, Actinomycetota, Bacteroidota, and Bacillota were also present, among others. Community composition was consistent between locations and across medusa structures (oral arm, bell, and gonad). The larval bacterial community clustered apart from the medusa community in beta diversity analysis and was characterized by the presence of several Pseudomonadota taxa that were not present in the medusa, including the *Alteromonas*, *Pseudoalteromonas*, and *Thalassobius* genera. A bacterial isolate library encompassing much of the amplicon sequencing diversity was also developed and tested via metabolic assays in a separate culture-dependent analysis of isolates from medusa bells, oral arms, and laplets. Most characteristics were not correlated with host sex or medusa structure, but gelatinase production was more common in laplet isolates, while lactose fermentation was more common in female oral arm isolates. The *Endozoicomonas* genus was dominant in both amplicon sequencing and in our isolate library, and was equally prevalent across all medusa structures and in both sexes. Understanding the bacterial component of the *C. xamachana* holobiont will allow us to further develop this important model cnidarian holobiont.

## Introduction

The upside-down jellyfish *Cassiopea xamachana* (Scyphozoa:Rhizostomeae) has been developed into a holobiont (i.e., a host and its associated microbiota) model system. Its photosynthetic dinoflagellates (Dynophycea: Symbiodiniaceae) play a dual role of nutrient source and metamorphosis inducer [1]. This mutualism is used to understand photosymbiosis in cnidarians, with applications towards coral conservation in mind. *C. xamachana* has produced pioneering work in nutritional symbiosis and symbiosis-driven development, while also being used in research on fluid dynamics, quiescence, and ecotoxicology (reviewed in [1]). However, little is known about the other symbionts of the *C. xamachana* holobiont: the bacteria and archaea. In scleractinian corals, these symbionts can play important nutritional and defensive roles in the holobiont, and coral microbiome composition can be an indicator of coral health and temperature tolerance [2–4]. One recent study investigated the bacterial community associated with a lab strain (T1A) of *C. xamachana* medusae and found that it consisted largely of members of Moraxellaceae and Pseudomonadaceae, and also indicated a role of these bacteria in nitrogen cycling [5]. However, laboratory strains of model species are known to associate with a markedly different microbial community compared to individuals in the wild [6,7]. Continued use of *C. xamachana* as a model to understand cnidarian symbiosis will be strengthened by an understanding of its endemic bacterial microbiome.

*C. xamachana* develops through a complex life cycle [8]. Adult gonochoric medusae brood aposymbiotic (lacking the photosymbionts) larvae which are released and settle on decaying mangrove leaves [9,10]. Once the larvae settle, they undergo metamorphosis to an aposymbiotic polyp [11]. Polyps will remain in this stage indefinitely until colonization by a member of the Symbiodiniaceae, typically *Symbiodinium microadriaticum* in the wild [12,13]. Upon colonization, *C. xamachana* undergoes a second metamorphosis, termed strobilation, during which the polyp will release a pelagic ephyra. The ephyra will swim off and mature into an adult medusa, which will then alight on the sand in shallow marine waters, positioned oral side up in such a way to collect enough light for photosynthesis by the Symbiodiniaceae [11,14]. Bacteria from the *Vibrio* and *Pseudoalteromonas* genera are known to be important in *C. xamachana* larval settlement and larval metamorphosis [15,16]. Across developmental stages, the bacterial community is known to vary in non-photosymbiotic jellyfish [17]. A change in microbiome can have important phenotypic implications for the respective life stage [18–22]. Characterizing the *Cassiopea* developmental microbiome will aid our understanding of how bacteria contribute to the photosymbiosis and physiology of this holobiont.

**Fig 1:**
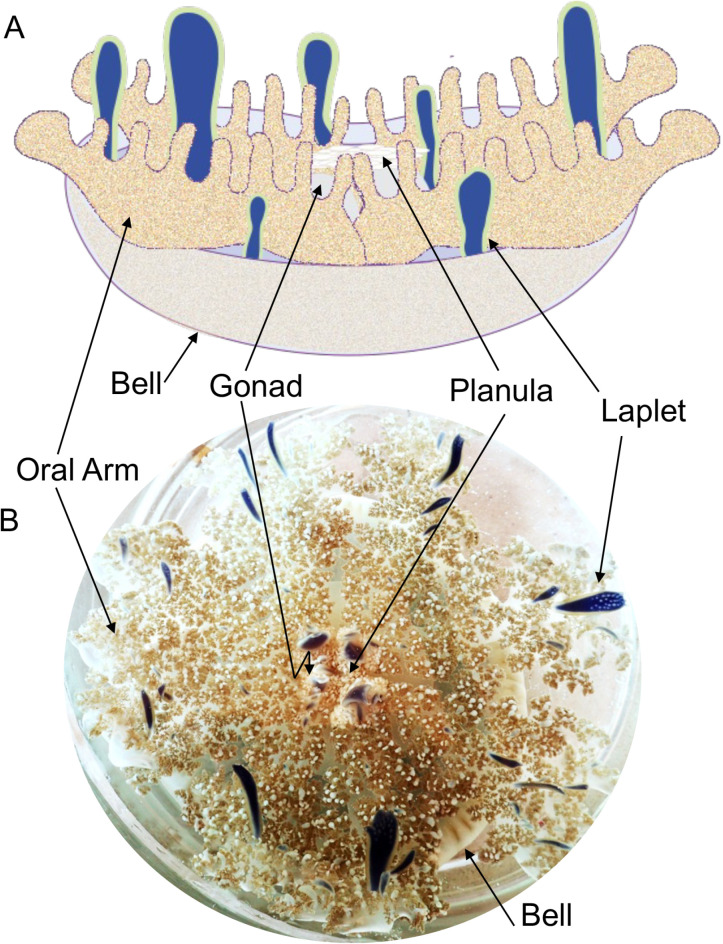
Lateral diagram (A) and oral surface photograph (B) of a *C. xamachana* medusa. Note that the oral surface of *C. xamachana* will usually be oriented towards the water surface to collect sunlight. The muscular bell contracts to increase water flow over the oral surface and help the oral arms collect food from the water, while the laplet function remains uncharacterized. Gonads are subsurface structures and cannot be viewed from the oral surface.

Here we examine the bacterial community associated with the larval and adult medusae stages. We look at various structures associated with the adult medusa, including the bell, oral arm, gonad, and laplet (Fig 1). The muscular bell’s pumping motion forces water to flow over the oral arm which can then collect food from the water column. The laplet, also known as a foliaceous appendage, vesicular appendage, vesicle, or oral appendage, is a brightly colored elongated structure found sporadically on the oral surface of *C. xamachana* (Fig 1). The function of the laplet is undescribed to date, but these structures range in color from yellow-green to deep blue [23,24].

This study presents the first examination of the adult and larval bacterial fraction of the *C. xamachana* holobionts sampled in the wild. Culture-independent methods for sequencing the microbiome provide a holistic view of community composition, without the worry of missing fastidious microorganisms or rarer members of the community [25,26]. In culture-independent characterization of the holobiont, adult medusae were collected from two sites, and bell, arm, and gonad samples, in addition to larvae, were examined separately to determine whether the bacterial community is consistent across populations in the Florida Keys, across various medusa structures, and across life stages. Furthermore, in a separate culture-dependent examination of the microbiome, bacterial cultures were isolated from adult medusae bells, oral arms, and laplets. Culture-dependent methods are still useful in learning about symbiosis function, as well as microbe-microbe and host-microbe interactions [27]. Bacterial isolates were identified and metabolically characterized to gain insight into the functional profile of the bacterial microbiome and whether that varies by host sex or structure. Culture-independent analysis revealed a bacterial community consistent across the oral arm, bell, and gonad, but distinct between life stages, while culture-dependent analysis demonstrated that bacterial metabolic capacity was largely consistent across the oral arm, bell, and laplet, and in male and female jellyfish.

## Materials and methods

### Bacterial community composition: Collection

Adult jellyfish were collected from Long Point Key (24°45’04.7”N, 80°58’43.5”W, n=3) and Key Largo (25°06’05.6”N, 80°26’19.9”W, n=3) in 2014. No permit was required for this study which complied with all relevant regulations. Collection of up to 10 *Cassiopea* over two days is allowed per individual according to the Florida Fish and Wildlife Conservation Committee (https://myfwc.com/). Long Point Key jellies (2 males, 1 female) were collected from a depth of 2 m, while Key Largo jellies (3 males) were at less than 1 m depth. Upon collection, jellyfish from Long Point Key were placed in a cooler in seawater collected from the same site and transported to Key Largo, where they were placed in aquaria and sampled within two hours. Jellyfish from Key Largo were scooped from the water with a fish net and immediately placed into a flow-through tank with seawater from the same site. After tissues were sampled from each individual, jellyfish were immediately returned to the water. Animals from Long Point Key were always kept separate from those from Key Largo. Samples were collected from the bells (n=5), gonad (n=5), and oral arms (n=8), within 2 hours of collection (Fig 1). Gonad samples were dissected from the individual using sterilized forceps to pull tissue from the subgenital pits. All gonad samples were from male jellyfish. Approximately a 0.5 cm^2^ tissue sample was collected for bell and oral arm samples. Two oral arm samples were collected from each of two jellyfish, one oral arm sample was collected from each of the other four jellyfish. Tissue samples were rinsed in filter-sterilized artificial seawater to remove transient and contaminating bacteria and immediately frozen in a dry shipper and stored at -80 °C until further processing.

Larvae were collected from brooding females at the Key Largo site (n=3) in 2014. Egg masses (Fig 1) were gently detached from the brooding vesicles using a sterile glass pipet to direct a jet of water at the base of the vesicles where the egg/embryos were attached. The released egg masses were collected into 0.2 µM filtered artificial seawater (ASW, Instant Ocean, Blacksburg, VA). Larvae were allowed to hatch overnight and rinsed with 0.2 µM filtered ASW prior to collection. Approximately 100–200 larvae were collected for each female and later all collected larvae from a given female were pooled for extraction. Larvae were immediately frozen in a dry shipper and stored at −80 °C until further processing.

### Bacterial community composition: Next generation sequencing analysis

Tissue samples were homogenized in lysis buffer with a sterile plastic pestle. Bacterial DNA was extracted from adult jellyfish structures and larvae samples using the MoBio PowerSoil DNA Isolation Kit (QIAGEN, Germantown, MD). The V4 region of the 16S rRNA gene was amplified using 515F and 806R primers (underlined) with linker sequences at the 5’ ends: (5’- A CAC TGA CGA CAT GGT TCT ACA GTG CCA GCM GCC GCG GTA A -3’) and (5’- T ACG GTA GCA GAG ACT TGG TCT GGA CTA CHV GGG TWT CTA AT -3’) respectively [28]. Amplified segments were sequenced on an Illumina MiSeq sequencer (Illumina, San Diego, CA, USA) by the DNA Services Facility at the University of Chicago, Illinois. While this primer pair was designed to capture bacterial and archaeal diversity, it underestimates Crenarchaeota and Thaumarchaeota archaeal diversity [29,30] so archaeal results should not be used to infer absence of taxa. While no primer pair is truly universal for bacterial diversity, this pair performed well when compared to others and led to the highest diversity estimates in one analysis [31], although it is biased against the Alphaproteobacterial SAR11 clade [32].

Processing of the 16S sequences was performed using QIIME1 (v1.9) [33]. Chimeric sequences were removed using USEARCH 6.1, and OTUs (operational taxonomic units) were selected at 97% similarity using UCLUST. All samples were rarified to 6000 sequences. Sequences identified as chloroplasts were removed. Analysis of similarities (ANOSIM) analyses were conducted and NMDS plots of Bray-Curtis beta-diversity analyses were created in R using the VEGAN package [34]. Alpha diversity analyses using the Chao1, Phylogenetic Diversity, and Shannon equitability metrics were completed in QIIME1 and visualized in GraphPad Prism (v9.5.1). Unpaired t-test analyses of alpha diversity across life history stage and one-way ANOVA of alpha diversity across structure types were also completed in GraphPad Prism (v9.5.1). The combined OTU and taxa assignment table are provided (S1 Table), as is a fasta file of representative OTU sequences (S1 File). Sequences were deposited in the NCBI Sequence Read Archive (SRA) with associated metadata as accession numbers SRR29499237-SRR29499257, under BioProject PRJNA1127054.

### Bacterial metabolic profiling: Collection and bacterial isolation

Male (n=3, 15 cm, 17 cm, 13.5 cm) and female (n=3, 18 cm, 17 cm, 10 cm) adult jellyfish were collected from <1 m depth in Key Largo (25°06’05.6”N, 80°26’19.9”W, n=3) in May 2019. Jellyfish were kept in seawater from the same site until tissue samples were obtained within 30 minutes of collection. Samples were collected from the bell, oral arm, and laplet (Fig 1) using ethanol-sterilized scissors and tweezers, and surface sterilized in 70% ethanol. The tissue was cut into small pieces with an ethanol-sterilized razor and homogenized with a plastic pestle in 0.22 µm filter-sterilized seawater. Homogenate was plated on salt water tryptone agar with 0.3% glycerol [35] at 10^−2^ through 10^−5^ dilutions and incubated at room temperature for 48–96 hours. A morphologically diverse set of colonies (based upon colony color, texture, diameter, and margin) was picked for each sample and streaked for isolation (n=115). Morphological diversity was used to capture as much taxonomic diversity as possible. The first character of an isolate’s code (Fig 4) represents male (M) or female (F) jellyfish, the second character represents arm (A), bell (B), or laplet (L), and the third character indicates jellyfish individual. A total of 24 female arm, 17 female bell, 19 female laplet, 24 male arm, 13 male bell, and 18 male laplet isolates are stored in 25% glycerol at −80°C.

**Fig 2:**
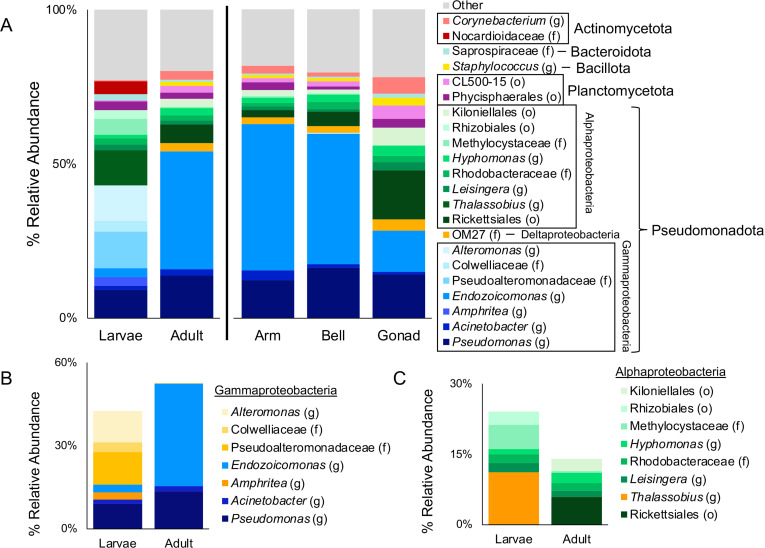
Bacterial community composition as determined by the V4 region of the 16S rRNA gene. The bacterial community was dominated by Pseudomonadota in all sample types (A). However, the taxa within the Gammaproteobacteria (B) and Alphaproteobacteria (C) varied between larval and medusa samples (taxa unique to larvae highlighted in yellow in B and C). Please note the varying Y axes in B and C. The “Other” category (A) includes taxa present at less than 2% average relative abundance in all 5 categories, including members of the Betaproteobacteria class, and the Gracilibacteria, Balneolota, Chlamydiota, Spirochaetota, Cyanobacteriota, and Chloroflexota phyla, which were just below this threshold. The Parvarchaea class of Euryarcheaota is also included in the “Other” designation. o: order, f: family, g: genus.

**Fig 3:**
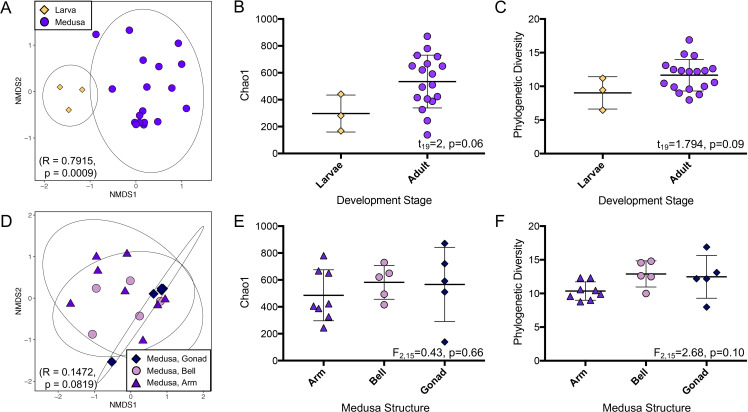
Alpha and beta diversity analyses of the *C. xamachana* bacterial community. Bacterial community composition varied between larva and medusa samples (A), but not between medusa structures (gonad, bell, arm, D) when analyzed via the Bray Curtis beta diversity metric. ANOSIM results are included in parentheses in NMDS plots (A, D). Larvae and medusae had similar alpha diversity as evaluated by unpaired t-test analyses of the Chao1 (B) and Phylogenetic Diversity (C) metrics. Medusa alpha diversity did not vary by medusa structure as evaluated by one-way ANOVA of the Chao1 (E) and Phylogenetic Diversity (F) metrics.

**Table 1:** NCBI reference sequences used in phylogenetic tree to identify isolates. All included taxonomic identifications are based on NCBI published records.

Phylum	Class	Order	Family	Species	Accession Number [Ref]
Pseudomonadota	Alphaproteobacteria	Caulobacterales	Hyphomonadaceae	*Henriciella algicola*	NR_157788 [37]
		Hyphomicrobiales	Stappiaceae	*Pseudovibrio denitrificans*	NR_029112 [38]
		Rhodobacterales	Roseobacteraceae	*Roseivivax sediminis*	NR_109179 [39]
				*Sulfitobacter faviae*	NR_152065 [40]
	Gammaproteobacteria	Oceanospirillales	Endozoicomonadaceae	*Endozoicomonas atrinae*	NR_134024 [41]
			Halomonadaceae	*Halomonas janggokensis*	NR_042489 [42]
			Oceanospirillaceae	*Bermanella marisrubi*	NR_042750 [43]
				*Oceaniserpentilla haliotis*	NR_042641 [44]
			Saccharospirillaceae	*Saccharospirillum mangrovi*	NR_179766 [45]
		Vibrionales	Vibrionaceae	*Vibrio natriegens*	NR_113786 [46]
				*Vibrio viridaestus*	NR_179904 [47]
Bacillota	Bacilli	Bacillales	Bacillaceae	*Bacillus sinesaloumensis*	NR_147383 [48]
				*Bacillus wiedmannii*	NR_152692 [49]
				*Metabacillus endolithicus*	NR_145872 [50]
				*Peribacillus acanthi*	NR_179899 [51]
				*Pontibacillus salicampi*	NR_181279 [52]
				*Priestia aryabhattai*	NR_118442 [53]
			Planococcaeae	*Bhargavaea beijingensis*	NR_117988 [54]
			Staphylococcaceae	*Staphylococcus epidermidis*	NR_113957 [55]
Actinomycetota	Actinomycetia	Micrococcales	Kytococcaceae	*Kytococcus sedentarius*	NR_074714 [56]
Balneolota	Balneolia	Balneolales	Balneolaceae	*Balneola vulgaris*	NR_042991 [57]
Bacteroidota	Cytophagia	Cytophagales	Reichenbachiellaceae	*Marinoscillum pacificum*	NR_043917 [58]
	Flavobacteriia	Flavobacteriales	Flavobacteriaceae	*Tenacibaculum geojense*	NR_117983 [59]

### Bacterial metabolic profiling: Sanger sequencing analysis

Colony PCR of the 16S rRNA gene was completed for most bacterial isolates (n=96 of 115). A small amount of a colony was picked with a sterile toothpick and diluted in 100 µl molecular grade water, and then 2 µl of that dilution was added to a 25 µl PCR reaction using GoTaq (Promega Corporation, Madison, WI), 27F (5’- AGAGTTTGATCCTGGCTCAG - 3’), and 1492R (5’ – GGTTACCTTGTTACGACTT - 3’). The PCR parameters included an initial denaturation for 3 min at 95°C, followed by 30 cycles of 30 sec at 95°C, 30 sec at 55°C, and 90 sec at 72°C, and finally 10 min at 72°C. PCRs were verified on agarose gels, then cleaned with ExoSAP-IT (Thermo Fisher Scientific, Waltham, MA), and sequenced at the Penn State Sequencing Core on a 3730XL Genetic Analyzer (Applied Biosystems).

**Fig 4:**
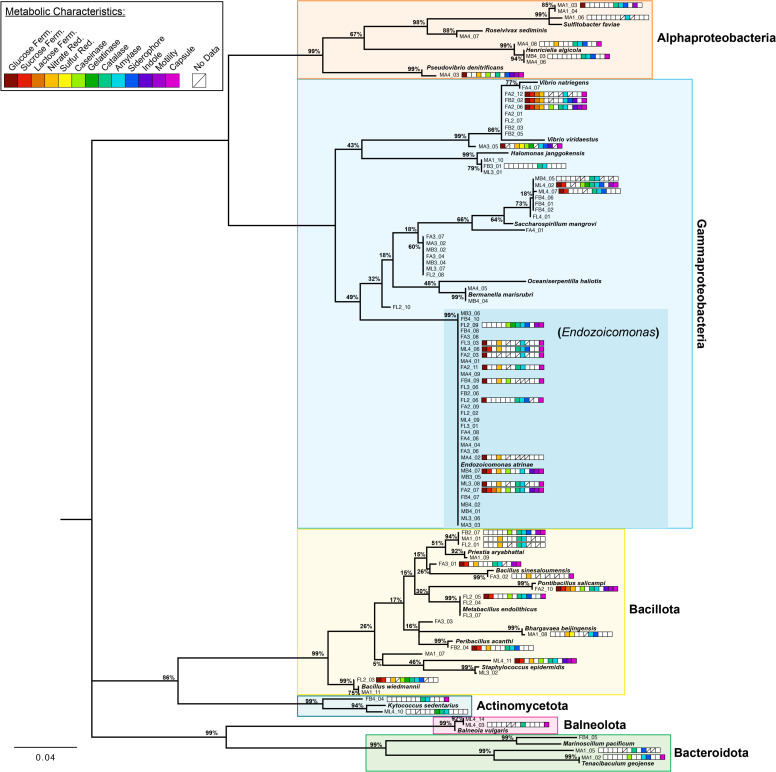
Maximum likelihood unrooted phylogenetic tree of 16S rRNA gene from *C. xamachana* bacterial isolates and reference sequences. Bootstrap values based on 100 replicates. Clade coloration indicates larger bacterial groups, either class or phylum. Metabolic characteristic results indicated by colored boxes next to the isolates selected for that study, legend at upper left. A white box indicates a negative result for the test, a box with a slash indicates no data was collected for that test. First character of isolate code represents male (M) or female (F) jellyfish, second character represents arm (A), bell (B), or laplet (L) structure, and third character indicates jellyfish individual. Bolded species names are reference sequences from NCBI (see Table 1). Bottom left scale bar indicates genetic distance.

Sequences were trimmed and quality checked in 4 Peaks v1.8, then taxonomic identity was assigned via Nucleotide BLAST with the 16S ribosomal RNA sequence (Bacteria and Archaea) database at the National Center for Biotechnology Information (NCBI) website. For all isolates, forward and reverse sequences were run through BLAST separately and compared for consistency. Initial taxonomic identity was based on the top BLAST result with the lowest E-value and highest percent identity. NCBI was used to identify similar reference sequences for taxa in the database to these initial taxonomic identifications in July 2023, which were downloaded and included in the analysis (Table 1). All reverse sequences were aligned via MUSCLE using the UPGMA cluster method in MEGA 11 [36]. A maximum likelihood phylogeny was constructed from the alignment using the Tamura-Nei model, uniform rates among sites, and the nearest-neighbor-interchange heuristic method with 100 bootstraps, also in MEGA 11. No outgroup was used due to the number of bacterial families represented. The phylogeny (Fig 4) was visualized in FigTree v1.4.4 (http://tree.bio.ed.ac.uk/software/figtree/), and taxonomic identifications were based on cluster patterns from this phylogenetic analysis. Sequences were deposited in the NCBI GenBank under accession numbers PP939074-PP939169.

### Bacterial metabolic profiling: Metabolic characterization

A subset of bacterial isolates was arbitrarily selected for further characterization, representing isolates from 20 male and 20 female *C. xamachana* medusae, including 9 bell, 18 oral arm, and 13 laplet isolates. We examined a variety of metabolic characteristics, including fermentation properties, diverse enzymes, whether a capsule was present, and motility. All metabolic characterization assays were completed in triplicate for each isolate to lower the risk of contamination skewing results. If an isolate did not have consistent growth or results across all three replicates then it was tested again, and if results and/or growth remained inconsistent it was removed from that analysis. All media was modified for marine bacteria through the addition of 2% sodium chloride. Unless otherwise noted all cultures were incubated at 28°C.

Fermentation of glucose (n=40), sucrose (n=40), and lactose (n=39) was tested with a phenol red pH indicator for acid production and a Durham tube for gas production, at a 0.5% sugar concentration [60]. Nitrate reduction capacity (n=38) was tested through growth in nitrate broth, and the subsequent addition of Remel Nitrate Reagents A and B (Thermo Fisher Scientific, Waltham, MA) once growth had occurred [61]. A red color change indicated the presence of nitrate reduction to nitrite. If no color change occurred a pinch of zinc powder was added to determine whether nitrate was still present [61]. If the media remained colorless, the reduction of nitrate to nitrogen gas was indicated, which was confirmed by the presence of gas in the Durham tube. Sulfide indole motility (SIM) medium (Thermo Fisher Scientific, Waltham, MA) was used to detect sulfur reduction and indole production (n = 37) [62]. Sulfur reduction was indicated by a dark coloration of the medium, while indole production was tested through the addition of Kovac’s reagent (Carolina Biological Supply Company, Burlington, NC) and a subsequent red color change [62].

Flagellar motility was examined via motility soft agar and 0.005% triphenyltetrazolium chloride (n=28), which turns red in response to metabolic activity by bacteria [62]. Red coloration away from the inoculation stab indicated a motile organism. Motility data were confirmed by the SIM medium results (above), which also indicated motility through the appearance of turbidity away from the inoculation stab (n=35).

Production of the amylase enzyme (n = 38) was examined through growth on starch plates [63]. Once cultures had grown, plates were flooded with iodine for several minutes to check whether starch had been degraded [63]. Isolates were grown on 5% nonfat milk agar plates to test for caseinase activity (n = 27) (adapted from [64]). A clearing surrounding isolate growth indicated digestion of the milk proteins. Gelatinase activity was tested through growth of isolates on 12% gelatin medium at 25°C (n = 40) [65]. Once growth had occurred tubes were refrigerated and checked for liquidity [65], which indicated that the gelatin had been digested. Catalase activity was examined in isolates grown on salt water tryptone agar with 0.3% glycerol (n=31). Three percent hydrogen peroxide was added to bacteria smeared on a glass slide with a sterile toothpick, and bubble production indicated that catalase was present [66]. Isolates were also grown on chrome azurol S (CAS) media modified for marine bacteria as previously described [67], with 4% glucose, 6% casamino acid, and 1mM FeCl_3_ (n=29). Siderophore production was determined by an orange clearing around the bacterial growth, indicating that siderophores had sequestered the iron present in the medium.

To examine whether bacterial cells produced capsules (n=40), cultures were grown overnight in seawater tryptone broth with 0.3% glycerol. A loop of culture was smeared on a slide and mixed with a drop of Congo red dye (Thermo Fisher Scientific, Waltham, MA) and air dried [68]. Samples were stained with Maneval solution (Carolina Biological Supply Company, Burlington, NC) for five minutes, rinsed with deionized water and again let air day [68]. Cells were examined at 1000x magnification for a white halo around individual bacterial cells, which indicated the presence of a capsule.

Metabolic characterization results were analyzed through Pearson’s Chi-squared test to compare structure types, host sex, and both structure type and host sex using the tidyr package in R [69]. Chi-squared results should be used with caution given the small sample size. Visualization of data was completed in R, using the ggplot2 [70] and ggmosaic packages [71]. A principle component analysis (PCA) of metabolic characteristics was visualized in R using the ggbiplot package [72].

## Results

### Bacterial community composition

The *Cassiopea xamachana* microbiome was species diverse, consisting primarily of members of the Pseuodomonadota (previously Proteobacteria; including the Alphaproteobacteria, Deltaproteobacteria, and Gammaproteobacteria classes), Planctomycetota, Bacillota (previously Firmicutes), Bacteroidota (previously Bacteroidetes), and Actinomycetota (previously Actinobacteria) phyla (taxonomy changes described in [73]). These phyla accounted for 93.47% of the average community (Fig 2A). No archaeal sequences were detected in the larval samples, and only trace amounts of two Parvarchaea taxa were found occasionally in medusa samples (averaging 0.15% of the community).

Larvae had a distinct bacterial/archaeal community when compared to medusae (ANOSIM: R = 0.7915, p = 0.0009, Fig 3A). Most bacterial classes were found in both larvae and adult medusae (Fig 2A), but Bacilli (Bacillota) and Deltaproteobacteria (Pseuodomonadota) were unique to medusae samples, while the Nocardioidaceae family (Actinomycetota) and *Amphritea* genus (Gammaproteobacteria) were unique to the larvae. Several Gammaproteobacteria taxa were more relatively abundant in the larvae than the medusae, including the Colwelliaceae and Pseudoalteromonadaceae families, and the *Alteromonas* genus (3.55%, 11.61%, and 11.35% in larvae vs 0.04%, 0.03%, and 0.05% in medusae, Fig 2B). Similarly, within the Alphaproteobacteria, the Rhizobiales order (including the Methylocystaceae family), and *Thalassobius* genus were much more relatively abundant in the larvae (7.83% and 11.07% in larvae vs 0.48% and 0.01% in medusae, Fig 2C). Few Pseudomonadota were better represented in the medusae. However, the Ricketsialles order (5.86% in medusae vs 0.11% in larvae), and the *Endozoicomonas* genus (36.98% in medusae vs 2.87% in larvae, Fig 2B) were present at higher abundance in medusae than in larvae. While the average alpha diversity was lower for the larval stage than the adult stage, this difference was not significant for either the Chao1 metric (t_19_=2, p=0.06, Fig 3B) or the Phylogenetic Diversity metric, (t_19_=1.794, p=0.09, Fig 3C). The Shannon Equitability metric was also tested, and in this metric the average was higher for the larvae than for the medusae, but again this difference was not significant (t_19_=1.534, p=0.14, S1A Fig). No clustering pattern was observed within the medusae samples by individual jellyfish (ANOSIM: R = 0.2332, p = 0.0514), nor by collection site (ANOSIM: R = -0.1214, p = 0.9074) using the Bray-Curtis metric (S2 Fig).

The bell, oral arm, and gonad communities sampled from adults did not exhibit clustering by structure (ANOSIM: R = 0.1472, p = 0.0819, Fig 3D). The possible exception is the gonad community, where four of the five samples clustered very tightly together in the NMDS visualization (Fig 3D). However, more sampling will be necessary in the future to confirm this observation. The arm and bell communities were remarkably consistent, both in composition and relative abundance. The gonad community was similar in composition but had a more even distribution of bacterial classes: notably much lower relative abundance of the *Endozoicomonas* genus (12.44% in the gonads vs 43.83% in bell/arms), and much higher relative abundances of the Rickettsiales, Kiloniellales, and Streptophyta orders (14.66%, 5.3%, and 3.32% in the gonads vs 3.39%, 1.48%, and 0.99% in bells/arms), as well as the CL500–15 order from the Planctomycetota (4.04% in the gonads vs 1.51% in bell/arms), and the *Corynebacterium* genus from the Actinomycetota (4.99% in the gonads vs 1.94% in bell/arms, Fig 2A). No difference was noticed in alpha diversity between structures when using the Chao1 metric (F_2,15_=0.43, p=0.66, Fig 3E), the Phylogenetic Diversity metric (F_2,15_=2.68, p=0.10, Fig 3F), or the Shannon Equitability metric (F_2,15_=0.65, p=0.54, S1B Fig).

### Bacterial metabolic profiling

Our culture collection captured much of the broad phylum and class diversity found in our community characterization (see Figs 2 and 4). Sanger sequencing of isolates (n=96) revealed members of the Alphaproteobacteria, Gammaproteobacteria, Actinomycetota, Bacillota, Balneota, and Bacteroidota (Fig 4). Members of the Deltaproteobacteria and Planctomycetota are currently missing from the sequenced culture collection; more targeted culture conditions may be able to add those in the future. Isolates included for metabolic characterization (n=40) included members of the Alphaproteobacteria, Gammaproteobacteria, Actinomycetota, Bacillota, Bacteroidota, and Balneolota (previously considered part of the Bacteroidota).

Metabolic capabilities of the isolates varied (Fig 4). A majority could ferment glucose (60% positive, n=40) but not lactose (13% positive, n=39) or sucrose (36% positive, n=39). A majority could reduce nitrate (61%, n=38) but very few could reduce sulfur (5%, n=37). Most exhibited amylase (89%, n=38) and catalase (90%, n=31) activity, and a majority showed siderophore production (62%, n=29). Some exhibited caseinase activity (52%, n=27), but few demonstrated gelatinase activity (13%, n=40) or indole production (22%, n=36). A minority were motile (31%, n=36), but a majority had a capsule (68%, n=40).

**Fig 5:**
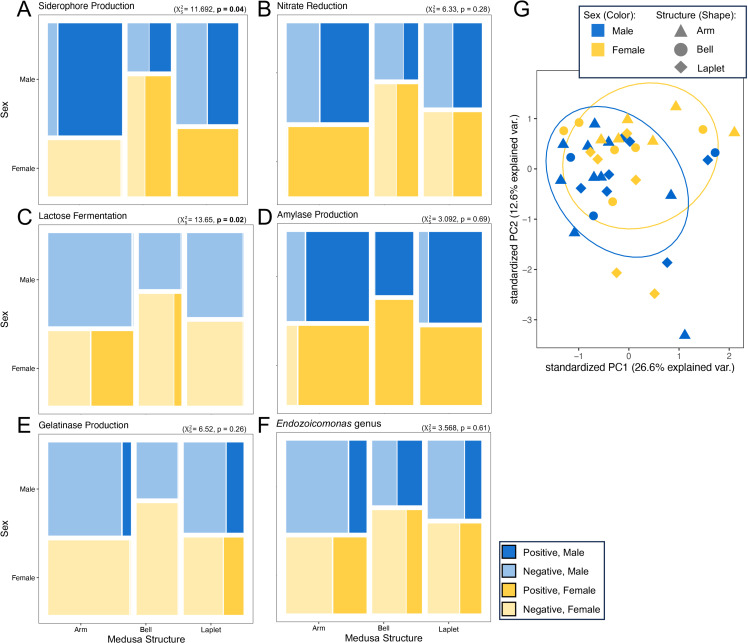
**Mosaic plots showing the distribution of isolates testing positive (darker shade) or negative (lighter shade) for a given characteristic, divided by sex (Y axis: male in blue, female in yellow) and by medusa structure (X axis).** Five metabolic characteristics were tested (A: siderophore production; B: nitrate reduction; C: lactose fermentation; D: amylase production; E: gelatinase production), and the distribution of isolates identified as members of the *Endozoicomonas* genus was also examined (F). Chi-squared results based on sex and structure groups are shown. Lactose fermentation was significantly more common in females than males when only sex was considered (${Χ}_{1}^{2}$ = 5.449, **p** = 0.02), while gelatinase production was significantly more common in laplets than in arms or bells (${Χ}_{2}^{2}$ = 6.046, **p** = 0.049). A principal component analysis of isolate metabolic characteristics (those shown in A-F, as well as sulfur reduction, capsule presence, motility, glucose and sucrose fermentation, indole production, caseinase production, and catalase production) demonstrated no clustering by sex (color, 95% confidence intervals) and/or structure (shape, G).

Isolate characteristics were analyzed by sex and structure to determine whether the bacterial metabolic profile varied by host origin. While Pearson’s Chi-Squared test should be interpreted here with caution due to limited sample sizes in the comparative groups, the ability to ferment lactose was associated with isolates from females (Χ^2^_1_ = 5.449, p=0.0196, Fig 5C), while siderophore production was most common in female laplet and male arm isolates and least common in female arm isolates (Χ^2^_5_ = 11.692, p= 0.0393, Fig 5A). Gelatinase activity was most common in laplet isolates, regardless of sex (Χ^2^_2_ = 6.046, p=0.0487, Fig 5E). No other metabolic property correlated with structure and/or sex (Fig 5,S2 Table, and S3 Fig). The lack of correlation between metabolism and isolate source is reflected in the PCA visualization, in which little separation is found between medusa structures, nor between the sexes (Fig 5G).

One third of the isolates (33 of 96) were identified as members of the *Endozoicomonas* genus. Eleven of the isolates included for metabolic characterization were identified as *Endozoicomonas* spp. and demonstrated variation in their metabolic capacity (Fig 4). Most could ferment glucose (91%, n=11) but not lactose (9%, n=11) or sucrose (27%, n=11). A majority could reduce nitrate (80%, n=10) but none could reduce sulfur (0%, n=11). Many exhibited caseinase (67%, n=6) and catalase (86%, n=7) activity, but not gelatinase activity (9%, n=11) or indole production (18%, n=11). All exhibited amylase activity (100%, n=9), but siderophore production was present in only half of those tested (50%, n=6). Few were motile (27%, n=11), and most had a capsule (91%, n=11). *Endozoicomonas* isolates were evenly distributed between sexes and across structures (Χ^2^_5_ = 3.568, p= 0.6131, Fig 5F, S2 Table).

## Discussion

*Cassiopea xamachana* has recently come under renewed interest for its potential as a cnidarian holobiont model system. However, while the *C. xamachana* – Symbiodiniaceae symbiosis has been well-explored (reviewed by [1]), little is known about other microbes present in this jellyfish. Here we demonstrate that the *C. xamachana* bacterial microbiome is similar to that found in other cnidarian holobionts (e.g., reef building corals), but appears less diverse, including hundreds of species instead of the thousands of species found in many tropical corals [74]. Cnidarians typically contain Archaea from the Nanoarchaeota and Crenarchaeota phyla [74] but only members of the Euryarchaeota were detected in *C. xamachana*, probably due to limitations of the primer set used [29,30], thus archaeal absence should be interpreted with caution. These sequences were identified as primarily from the Parvarchaea class and averaged only 0.16% relative abundance. Euryarchaeota have previously been found to be members of the mucus community associated with reef-building corals [75,76] and Parvarchaea was identified as a core community member of the coral *Platygyra daedalea* [77]. Within the Bacteria, most of the taxa present in *C. xamachana* are also common in cnidarians. We did not identify any *Propionibacterium* sp. or *Pelomonas* sp. sequences, recently included as members of core communities in healthy cnidarian tissues, but other members of the core were all present at various levels of relative abundance, including *Endozoicomonas* sp., *Pseudomonas* sp., Pseudoalteromonadaceae, *Alteromonas* sp., Vibrionaceae, and Sphingomonaceae (Fig 2) [74]. Pseudoalteromonadaceae and *Alteromonas* sp. were unique to larval samples. Pseudoalteromonads are known for antimicrobial-production [78,79], and could potentially be involved in larval defense against biofouling and/or pathogens. Bacterial defense of eggs has been found in other systems, including hydra, lobsters, shrimp, squid, and many insects [80–84].

The *Endozoicomonas* genus was a dominant taxon in adult *C. xamachana* samples, and was also present at low abundance in larvae (avg. 2.9%). The presence in both life stages could indicate a conserved role and vertical transmission of this group. While found in all structures, this genus occurred at much lower relative abundance in the gonad (avg. 12.4%) than in the arm (46.9%) or bell (40.8%, Fig 2). *Endozoicomonas* is known to grow in large aggregations in coral tentacles [85], potentially explaining the high relative abundance in our amplicon sequencing and the large proportion of our culture collection. However, the high relative abundance of this group in bell samples suggests that in *C. xamachana* these aggregations occur in the bell as well as in the oral arms. This localization is perhaps due to *Endozoicomonas* localizing to regions of high photosynthetic potential. *Endozoicomonas* isolates were cultured from the arms, bells, and laplets of both male and female jellyfish (Fig 5).

The genus *Endozoicomonas* has been divided into several clades and is thought to be functionally diverse [74,85]. *Endozoicomonas* isolates demonstrated diverse metabolic capabilities under laboratory settings, with closely related strains inconsistent in functional profiles (Fig 4). One proposed function of this genus in cnidarians is involvement in nutritional symbiosis, including nitrogen and sulfur cycling [5,85]. Most isolates were positive for nitrate reduction, supporting this putative functional role. Our assay found that reduction of nitrate to nitrite was common both among the *Endozoicomonas* genus and throughout the isolate collection, although one member of this clade (*Endozoicomonas* sp. MB4–07) was able to reduce nitrate to nitrogen gas (as was *Pseudovibrio* sp. MA4–03). Our *Endozoicomonas* isolates were unable to reduce sulfur. At least some *Endozoicomonas* spp. are thought to be an important part of a healthy coral microbial community [85], and finding them so dominant in *C. xamachana* provides more support for the use of this holobiont as a cnidarian model system.

The medusa bacterial community presented in this paper differs substantially from that previously described for the T1A lab strain of *C. xamachana* medusa [5]. Surprisingly, the bacterial community in that study consisted almost entirely of members of the Moraxellaceae and Pseudomonadaceae families. In our dataset these two families were represented almost entirely by the *Pseudomonas* [Moraxellaceae] and *Acinetobacter* [Pseudomonadaceae] genera, with minor amounts (0.08% average) of the Moraxellaceae genus *Enhydrobacter* present as well. While these groups are abundant in our samples, they average only 12.9% (Moraxellaceae) and 1.9% (Pseudomonadaceae) relative abundance, as opposed to >60% Moraxellaceae and 15–25% Pseudomonadaceae in the lab strain [5]. We were not able to culture members of these families in our isolate collection. Both families were also present in our larval samples (not tested in [5]), but at reduced relative abundance levels (Fig 2). Other families found by Röthig and colleagues [5] are present in our dataset, but only Rhodobacteraceae occurred at a substantive level in wild *C. xamachana* (averaging 5.03%). The differences between the lab-maintained T1A strain and the wild jellyfish bacterial communities may be due to host environment. Many marine invertebrate microbiomes shift when they are moved into aquaria. For example, the community composition of the tropical corals *Siderastrea siderea* and *Fungia granulosa* shifted extensively after transfer to aquaria [86,87], and similar patterns have been seen in squid and sea slugs [88,89]. Similarly, rodents experience a shift in their microbiome in a laboratory setting [7]. Marine invertebrate microbiome shifts were generally found to have occurred quickly (in one case in as little as a day [87]), and the *C. xamachana* T1A lab strain has been in culture for many years. The long-term laboratory maintenance of the TIA strain could thus be expected to explain the differences in bacterial microbiomes. This shift does not negate the functional importance of the microbiome. The overall function of the microbiome may be more importance than the taxonomic consistency of the microbiome, as per Doolittle’s *It’s the Song, Not the Singers* hypothesis [90,91]. Alternatively, certain selective pressures may be lacking or different in laboratory environments, changing the microbiome composition.

Many organisms exhibit distinct microbial communities as they move through development. Microbes can be acquired through vertical (parent to offspring) or horizontal (from the environment) transmission [92]. The overlapping but distinct community composition between larval and adult medusa stages (Fig 3) indicates that the microbiome may be the result of mixed transmission, with some members inherited from the parent and others acquired from the environment. Several taxa were unique to either larvae or adults (Fig 2), so the difference is not due solely to changes in relative abundance. The similar microbiome composition of adults collected from two sites approximately 40 miles apart indicates that some selective pressure may be involved in structuring the microbiome. The shift between larval and adult community composition could provide an additional explanation for the differences between the wild and lab communities. The *C. xamachana* T1A lab strain is generally maintained in the polyp stage (an intermediate stage between larvae and adult medusa which was not included in our study). If the polyp stage is also significantly different from the adult medusa stage, then the medusae would need to acquire new bacterial groups from their environment, which may lack bacterial diversity under laboratory conditions. The only study of the *C. xamachana* T1A lab strain did not include larval or polyp stages [5], but the differences in medusa bacterial composition discussed above suggests that all the life stages exist with less diverse holobionts. The microbial composition of lab seawater can vary substantially from environmental seawater, both in terms of reduced diversity and in the bacterial and archaeal taxa present [19,93]. Weiland-Bruaer [17] found lab-reared moon jelly *Aurelia aurita* polyps, ephyrae, and juvenile medusae differ in the composition of their bacterial communities. The ephyrae and medusae stages were associated with bacteria absent in the polyp stage [17], indicating that lab-reared animals can acquire stage-specific bacteria from the environment.

The bacterial metabolic profile did not vary substantially by host structure or sex (Fig 5, S2 Table, and S3 Fig), indicating that while the bacterial community is conserved between adults within the Florida Keys, little differentiation occurs within the jellyfish. While corals exhibit anatomical compartmentalization between skeleton, mucus, and tissue which have varying microbiomes [2], the basic body plan of the jellyfish lacks a skeleton entirely, and only the tissue community was examined here. Work on coral tissue has found a distinct microbiome in the coral gastroderm and in coral Symbiodiniaceae-colonized cells that varies from the overall tissue community [94]. A finer examination of the *C. xamachana* tissue could reveal variation between different tissue layers or the Symbiodiniaceae-associated amoebocytes. Alternatively, the ectoderm, endoderm, and mesoglea (a connective jelly-like middle layer) could provide similar environmental conditions for microbes throughout the jellyfish.

It is important to note that all bacterial isolates were tested under laboratory conditions, and that bacterial metabolic activity depends on environment. This study does not provide *in vivo* evidence for metabolic activity, but it does show the potential of bacterial isolates to utilize metabolic pathways in the jellyfish host. Within our isolate collection, gelatinase activity was found more in laplet bacterial isolates than in those from the bell or oral arms (Fig 5). Gelatin is present in fish and squid [95], and more importantly has been extracted from the jellyfish *Rhopilema hispidum* [96] and *Lobonema smithii* [97]. The higher prevalence of gelatinase bacteria in laplets could indicate that these structures contain more gelatin than the oral arms or bells.

Lactose fermentation was correlated with female oral arm bacteria (Fig 5). A hypothesis that the *lac* operon evolved to breakdown galactolipids into galactosyl-diglycerides [98] could provide insight. Galactolipids are a prevalent component of the thylakoid membrane in chloroplasts [99]. These bacteria may be opportunistically associated with jellyfish to access galactolipids. This association in turn could help prevent algal growth on the arms, or could help with digestion of algae and/or phytoplankton for food, as the breakdown of galactolipids in the chloroplasts could kill/help digest algae or phytoplankton settling on the arms or ingested by the mouths present on the oral arms. The five isolates with this capability come from three taxonomic groups: three are closely related to *Vibrio viridaestus* (*Vibrio* sp. FB2–02, FA2–06, and FA2–12), one is the single *Endozoicomonas* (*Endozoicomonas* sp. FA2–07) isolate studied here that could ferment lactose, and one is a member of the Bacillota phylum related to *Pontibacillus salicampi* (*Pontibacillus* sp. FA2–10).

Siderophore production was widespread in the isolate collection, missing only from female arms (and was ubiquitous in isolates from female laplets, Fig 5). Siderophores were not associated with any one phylogenetic group, but were widely distributed across our tree (Fig 4). Iron is an important component of the electron transport chain and various enzymes, but is limited in animal hosts [100,101]. Iron sequestration for symbiotic bacteria can be crucial for their growth, explaining the widespread distribution seen in our isolates. However, all isolates from female arms lacked siderophore activity. One explanation could be that if more iron is available in this microenvironment to support the production of eggs and larvae, siderophores might not be necessary, but further research is needed into this topic.

Amylase activity was prevalent throughout the isolate collection (Figs 4 and 5). The use of amylase to breakdown starch into sugars can be useful nutritionally. *C. xamachana* are mixotrophic, and feed on zooplankton, crustaceans, ostracods, chironomids, and nematode eggs and larvae [102,103] in addition to relying on translocation from their photosymbionts. Algae, which would contain starch that bacteria could breakdown with amylase, has also been found in the gut of wild *C. xamachana* medusa, although it was hypothesized to be accidental ingestion [103]. One form of amylase, α-amylase, has been experimentally used to modify Symbiodiniaceae surface molecules [104], and bacteria might also be able to use amylase to interact with the photosymbionts (Symbiodiniaceae). Alternatively, these bacteria may not use the amylase pathway while in symbiosis with the jellyfish host.

While this study is limited in scope, it provides a first look into the bacterial microbiome of wild members of this cnidarian holobiont model system. Future research should investigate the microbiome associated with wild and lab *C. xamachana* polyps. The culture collection described here should enable new research manipulating the bacterial microbiome associated with jellyfish to better understand the *in vivo* functional contributions. In this warming world, tropical corals are struggling. Understanding the interconnected dynamics between the cnidarian host, Symbiodiniaceae photosymbiont, and bacterial microbiome is crucial if we want conservation efforts to succeed. As corals bleach and experience disease outbreaks, model systems can be used to expedite discovery for translational applications for reef management, restoration and conservation. The overlap between the epibenthic lifestyle, nutritional Symbiodiniaceae symbiosis, and now the bacterial community similarity all make *C. xamachana* promising as one such model system.

## Supporting information

S1 Table Combined OTU and taxa assignment table for next generation sequencing analysis. (XLSX)

S2 Table Results of Pearson’s Chi-Squared test for bacterial metabolic tests and the presence of isolates from the *Endozoicomonas* genus. Significant p-values are bolded.
(XLSX)

S1 File Representative OTU sequence fasta file for next generation sequencing analysis. (FASTA)

S1 Fig Alpha diversity of the *C. xamachana* microbiome as measured by the Shannon Equitability metric did not vary between larvae and adults (A), nor between medusa structures (B). (TIF)

S2 Fig Medusa microbiome samples did not cluster by individual (A) or by collection site (B), indicating that overall bacterial community composition is consistent within a localized geographic region (spanning ~40 miles) and is conserved across the population. One individual was removed from the NMDS visualization since it was represented by only one sample, but it was included in the ANOSIM results.
(TIF)

S3 Fig Mosaic plots showing the distribution of isolates testing positive (darker shade) or negative (lighter shade) for a given characteristic, divided by sex (Y axis: male in blue, female in yellow) and by structure (X axis). Eight metabolic characteristics were tested (A: indole production; B: caseinase production; C: catalase production; D: capsule presence; E: sucrose fermentation; F: glucose fermentation; G: sulfur reduction; H: motility). Chi-squared results based on sex and structure groups are shown, Chi-squared tests were also performed for sex alone and structureti alone and no significant results were found for any of those tests (S2 Table).
(TIF)
