# Supplementary figures and images for: Cassiopea xamachana microbiome across anatomy, development, and geography
Source: PLoS One. 2025 Apr 11;20(4):e0319944. doi: 10.1371/journal.pone.0319944 (PMC11991732; doi:10.1371/journal.pone.0319944)

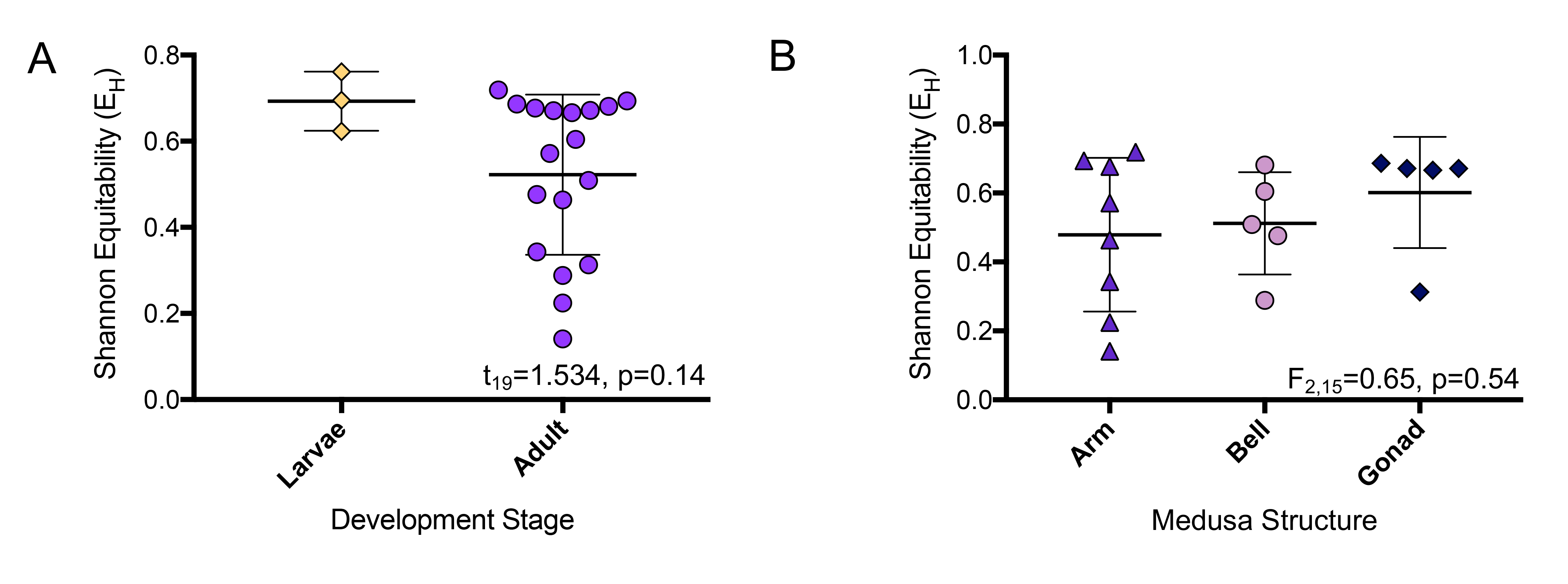

Supplement: S1 Fig — (TIF) [file pone.0319944.s004.tif]

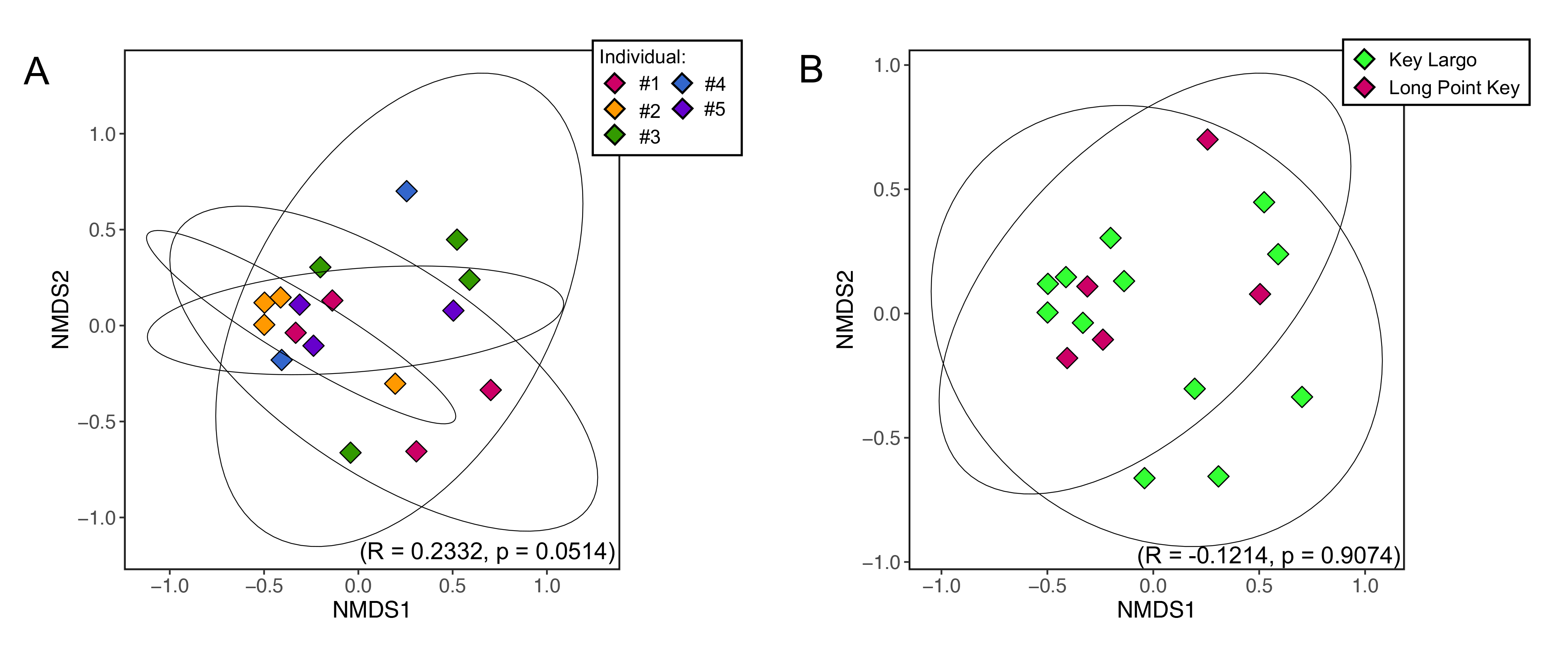

Supplement: S2 Fig — One individual was removed from the NMDS visualization since it was represented by only one sample, but it was included in the ANOSIM results. (TIF) [file pone.0319944.s005.tif]

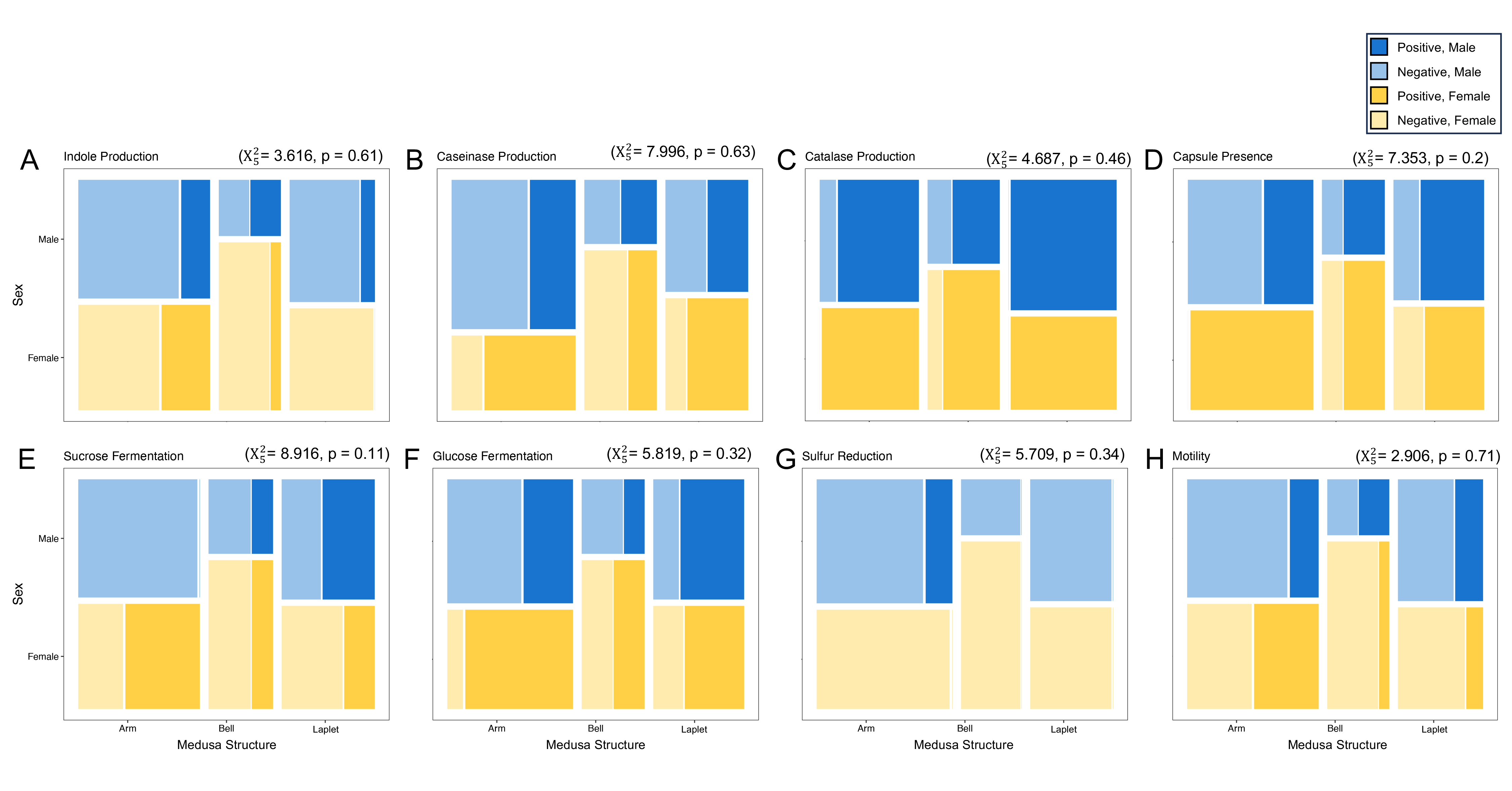

Supplement: S3 Fig — Eight metabolic characteristics were tested (A: indole production; B: caseinase production; C: catalase production; D: capsule presence; E: sucrose fermentation; F: glucose fermentation; G: sulfur reduction; H: motility). Chi-squared results based on sex and structure groups are shown, Chi-squared tests were also performed for sex alone and structureti alone and no significant results were found for any of those tests (S2 Table). (TIF) [file pone.0319944.s006.tif]
